# Is there a relationship between self-efficacy, disability, pain and sociodemographic characteristics in chronic low back pain? A multicenter retrospective analysis

**DOI:** 10.1186/s40945-019-0061-8

**Published:** 2019-10-12

**Authors:** Silvano Ferrari, Carla Vanti, Marta Pellizzer, Luca Dozza, Marco Monticone, Paolo Pillastrini

**Affiliations:** 1 Department of Biomedical Sciences, University of Padova, Padova, Italy; 2 Department of Biomedical and Neuromotor Sciences (DIBINEM), Alma Mater Studiorum, University of Bologna, Bologna, Italy; 3 Public Health Company - ULSS 7 Pedemontana, Bassano del Grappa, VI Italy; 4 Department of Experimental, Diagnostic and Specialty Medicine (DIMES), Alma Mater Studiorum, University of Bologna, Bologna, Italy; 5 Department of Medical Sciences and Public Health, University of Cagliari, Cagliari, Italy

**Keywords:** Pain self-efficacy, Spinal pain, Outcome measures, Disability, Musculoskeletal disorders

## Abstract

**Background:**

Pain-related self-efficacy is defined as the beliefs held by people with chronic pain that certain activities can be carried out despite the pain. Poor self-efficacy is an obstacle to the recovery and predicts long-term disability. The aims of this study are to investigate the prevalence of poor pain self-efficacy in Italian subjects with chronic low back pain (LBP), and to inquire the relationships between self-efficacy, disability, pain, and main demographic and clinical characteristics.

**Methods:**

A secondary multicenter retrospective analysis was done on 310 outpatients with chronic non-specific LBP. The pain self-efficacy measured with the Pain Self-Efficacy Questionnaire (PSEQ), the disability measured with the Roland & Morris Disability Questionnaire, and the pain intensity measured with the Numerical Rating Scale were considered variables to investigate, whereas demographic and clinical variables were considered predictors or potential confounders. A 40/60 PSEQ score was adopted as cut-off to distinguish between good and poor self-efficacy.

**Results:**

199 subjects (64.2% of the sample) showed poor self-efficacy. The odds of having poor self-efficacy appeared significantly related to female gender (OR = 1.80, 95%CI [1.12;2.90]; *p* = 0.015) and drugs use (OR = 1.68, 95%CI [1.06;2.70]; *p* = 0.029). Significant relationships also emerged between disability and higher age (β = 0.07, 95%CI [0.01; 0.12]; *p* = 0.02), being female (β = 1.80, 95%CI [0.32;3.29]; *p* = 0.018), low educational level (β = − 1.68, 95%CI [− 2.59;-3.29]; *p* < 0.001), higher height (β = − 0.08, 95%CI [− 0.158;-0.002]; *p* = 0.045), pain duration [mos] (β = 0.01, 95%CI [0.001;0.021]; *p* = 0.041), and drugs use (β = 2.86, 95%CI [1.44;4.27]; *p* < 0.001). The amount of pain appeared significantly related to educational level (β = − 0.47, 95%CI [− 0.76;-0.182]; *p* < 0.001), smoking (β = 0.56, 95%CI [0.09; 1.03]; *p* = 0.021), height (β = − 0.03, 95%CI [− 0.05; − 0.002]; *p* = 0.036), and drugs use (β = 0.81, 95%CI [0.399;1.22]; *p* < 0.001). No significant correlation appeared among weight, body mass index, and referred pain neither in relation to self-efficacy, nor in relation to pain/disability.

**Conclusions:**

The majority of our sample, composed of Italian people complained of chronic LBP, shows poor self-efficacy. Female gender and drugs use are significantly related to poor self-efficacy, low educational level negatively influences the amount of perceived pain and disability, and older age and smoking are related to disability and pain intensity, respectively. The knowledge of these sociodemographic and clinical characteristics potentially influencing chronic LBP may be useful to address more efforts towards the most negatively impacted subjects, among the entire population complained of chronic LBP.

## Background

Several studies in current literature stress the relationship between cognitive-behavioral factors and low back pain (LBP), pointing up the role of psychological dysfunctions in determining chronic pain or disability. Poor pain-related self-efficacy, fear of movement and catastrophizing may be relevant obstacles to recovery; nevertheless, they are potentially modifiable through clinical interventions, best-practice oriented [1].

According to the Fear-Avoidance Model described by Vlaeyen & Linton [2], pain catastrophizing, fear of movement and avoidance behavior might lead to physical deconditioning and pain perpetuation in chronic LBP. On the contrary, high pain self-efficacy appears a condition able to avoid this vicious circle [3].

Fear of movement (also called kinesiophobia) is a patien’s belief that certain activities should be avoided due to fear of provoking pain or re-injury [4]. It has been suggested that this condition predicts future disability in patients with LBP [2, 5]. Continuous avoidance behaviors are associated with depressive symptoms, higher pain intensity, disuse, and greater physical impairment and disability [2, 6]. Collectively, these studies support kinesiophobia as negatively influencing outcomes in individuals with LBP. Some authors also argue that fear-avoidance behaviors are the most specific and powerful cognitive factor in patients with LBP [7] and, in workers who claimed compensation for work-related back pain, it may be relevant in explaining failure to go back to work [8].

Pain catastrophizing is one of the most important psychological variables explaining pain responses. It has been defined as ‘an exaggerated negative orientation towards actual or anticipated pain experiences’ and explains a tendency to misinterpret or magnify apparently threatening situations [9]. It can lead to increased pain sensitivity, thus pulling patient in a vicious circle that may also reduce bodily performance. This condition has been well explained by the Fear-Avoidance Model described above, in which kinesiophobia is influenced by catastrophizing. People with high pain catastrophizing show worse pain outcomes, including increased pain, disability, and emotional distress [10]. Finally, higher levels of daily pain catastrophizing are related to higher daily pain intensity and negative metacognitive beliefs [11].

Pain-related self-efficacy is defined as the beliefs held by people with chronic pain that are able to carry out certain activities, even when experiencing pain [12, 13]. Previous studies showed self-efficacy beliefs as considerably influencing the rate of coping strategies use [14, 15], moderately correlating with pain intensity and strong associating with disability [16–18].

In patients with chronic LBP, pain self-efficacy is shown to be a significant mediator in the relationship between pain intensity and disability [19–21]. This role is considered more relevant than catastrophizing [21]. Poor pain self-efficacy is a more important obstacle to recovery than several other psychosocial factors in patients with LBP [22].

Patients with chronic LBP reporting high levels of pain self-efficacy also demonstrate higher levels of activity, working endurance, exercise/stretching performing [23], lower distress and severity of pain [24, 25], fewer maladaptive pain-related behaviours, lower catastrophizing [13], and greater use of various coping strategies (i.e., ignoring pain sensations, pacing) [13, 23]. On the contrary, weak self-efficacy predicts long-term disability [26, 27]. Self-efficacy and fear avoidance beliefs are more important predictors of disability than pain intensity and pain duration, in primary care patients with musculoskeletal pain [28].

Improvements in self-efficacy occurring during treatment are associated with better function and reduced self-reported pain [29, 30].

The relationship between the cited psychosocial factors and illness behaviours is the expression of an individual response to pain [1], but it may change due to different characteristics of the patients. Despite the correlations between psychosocial factors, disability and pain have been extensively studied, less knowledge is available on their relationship with socio-demographic characteristics, such as age, gender, work, education, etc.

In LBP patients older age seems predictive of poor treatment outcome [31]; male gender appears associated to worse fear avoidance score, particularly in under 50 years old patients complaining of heterogeneous chronic pain (47,9% of them with back pain) [32]. Unemployed people shows worse scores in psychosocial questionnaires, whereas employed people with chronic musculoskeletal pain have the best scores [32, 33]. Gender and income appear significant for self-efficacy and fear avoidance beliefs in chronic LBP patients [25], and obese people have moderately higher kinesiophobia and disability, compared to non-obese people with chronic LBP [34].

The scope of this study was to perform a secondary retrospective analysis of previous databases on chronic non-specific LBP in Italian subjects submitted to a conservative treatment and completing the Pain Self-Efficacy Questionnaire [13]. We aimed to investigate the prevalence of poor pain self-efficacy and to analyze the relationship between pain self-efficacy, lumbar disability, pain, and demographic and clinical characteristics.

## Methods

STROBE recommendations [35] for observational studies were followed.

### Study Design

Multicenter retrospective analysis.

### Setting

The medical records of outpatients with chronic non-specific LBP submitted to a conservative treatment in three physiotherapy clinics located in northern Italy were retrospectively reviewed. These records were already used for three previous studies on self-efficacy [15, 16, 36], so the present study design is secondary analysis.

### Population

The eligible patients for this secondary analysis were 372 subjects (165 from the study by Chiarotto and colleagues, 2015 [15]; 104 from the study by Chiarotto and colleagues, 2016 [36], and 103 from the study by Ferrari and colleagues, 2016 [16]. The centers involved in the previous studies are both public and private physical therapy clinics, chosen from different northern Italian regions, based on the clinicians’ availability to collect data. We excluded 62 records, which did not contain all the data required for the socio-demographical correlations, leaving a final sample of 310 outpatients with chronic LBP, who were retrospectively studied. Inclusion criteria concerned outpatients older than 18 years, complaining of chronic non-specific LBP with or without referred pain. Exclusion criteria were previous lumbar surgery, systemic diseases (inflammation, infection, cancer, etc.), neuromuscular disorders, or cognitive deficits. The clinical condition and inclusion criteria for the study were assessed by occupational unit clinicians, orthopaedic doctors or spinal surgeons, before referring the subjects to the physical therapy clinics. The patients’ enrolment was performed at the time of previous studies, ranging from March 2012 and April 2015.

The sample of the present, retrospective analysis was composed of 190 women (61.3%) and 120 men (38.7%), with a mean age of 49.83 ± 14.35 years and a median LBP duration of 12 months (IQR: 6–36). All demographic and baseline characteristics (sex, age, civil state, educational level, employment, Body Mass Index, smoke, pain location, pain duration, drugs consumption and comorbidities) and scales scores [Pain Self-Efficacy Questionnaire (PSEQ), Roland & Morris Disability Questionnaires (RMDQ), and Numerical Rating Scale (NRS)] were filled out during the first physical therapy session and were subsequently collected for each center in a predefined MS excel spreadsheet. Disability sub-investigation due to LBP, measured with the RMDQ questionnaire, was collected on a subgroup of patients consecutively included in the dataset. All spreadsheets have been pooled together at the analysis time for data quality assessment and statistical analysis.

### Measures

All participants filled in the PSEQ at the starting of their treatment period, to measure the degree of pain self-efficacy [13]. Each patient was asked to rate how confidently he/she can perform some activities, at present, despite his/her pain. In this study, the Italian version of the PSEQ (PSEQ-I) was used since it showed to be unidimensional, to display good internal consistency, reliability and construct validity, to have no floor/ceiling effects [15], and good responsiveness [36] in patients with chronic LBP. Each item is scored on a 7-point Likert scale, where 0 = not at all confident, and 6 = completely confident. Total scores are calculated by adding the score of each item, ranging from 0 to 60. Higher scores reflect stronger self-efficacy beliefs, whereas low scores indicate a subject more focused on his/her pain (seeking pain relief first).

PSEQ-I scores higher than 40 indicate a subject who is well responding to an exercise program [37] or sustaining and building on one’s own functional gains [13]. To facilitate the interpretation of data, in the present study we decided to classify PSEQ-I baseline scores as low (≤ 40/60), or high (> 40/60), similarly to a previous study on LBP [30].

The NRS was used to measure the subjective perception of pain, and the RMDQ for rating the disability level. The NRS is composed of 11 values ranging from 0 (no pain) to 10 (unbearable pain), indicating the main intensity of pain experienced by a patient in the last week. The NRS demonstrated good sensitivity and responsiveness [38]. A close association between changes on the Pain Intensity and the Patient Global Impression of Change was also demonstrated [39].

The RMDQ is a health status measure designed to be completed by patients to assess physical disability due to LBP. Patients completing the RMDQ are asked to place a check mark beside a statement if it applies to them. The RMDQ score is calculated by adding up the number of items checked, therefore it ranges from 0 (no disability) to 24 (maximum disability). The RMDQ demonstrated good psychometric properties, evidenced by internal consistency and responsiveness [40].

Pain self-efficacy measured with the PSEQ-I, pain intensity measured with the NRS, and disability measured with RMDQ were considered the variables to investigate, and assume the role of response variables, whereas the other variables (pain characteristics, psychosocial variables, etc.) were considered predictors or potential confounders. Two research assistants provided all the participants with written information concerning the questionnaires and procedures.

### Ethical considerations

Based on the study design, ethic approvals were previously obtained by the Institutional Review Board Operative Unit of Physical and Rehabilitation Medicine, Salvatore Maugeri Foundation, Scientific Institute of Lissone, Milan, Italy, and by the Ethics Committee of the University Hospital S.Orsola-Malpighi, Bologna, Italy. Consent forms had already been signed, and further forms were not required. The privacy rights of participants were observed and the procedures followed were in accordance with Italian ethical standards and with the Helsinki Declaration of 1975, as revised in 2000.

### Statistical analysis

Given the retrospective nature of this study, a formal power analysis was done only to avoid underpowered results at the time of the final statistical analysis. Considering the general linear models approach and accepting a type I alpha error of 0.05, a maximum number of predictors of 11 and a proportion of variation explained by the model between 10 and 15%, the calculated statistical power associated to a sample size of 310 observations was higher than 99%. All continuous variables were summarized using descriptive statistics and in particular were reported as mean ± standard deviation (SD) or median and its interquartile range as appropriate. No methods for handling missing values (NAs) was adopted, we did our best to obtain full completeness of each variable before analysis was begun. In case missing values persisted, absolute frequencies of NAs were reported but were not included in the relative frequencies calculation to avoid underestimated results.

Normal distribution was verified inspecting histograms and evaluating skewness and Kurtosis indexes. Categorical or dichotomous variables were reported with their absolute and relative frequencies. Crude prevalence rate as primary aim was reported for patients showing poor self–efficacy score results in respect to the entire sample analyzed.

Linear regression analysis was adopted to explore the correlation between the PSEQ-I, NRS and RMDQ with demographic and baseline characteristics. Multivariable analysis according multiple linear regression model were considered in order to obtain the predictive set of independent variables affecting the outcome score. In order to have estimates of the Odds Ratios related to baseline demographic characteristics and PSEQ-I score, a logistic regression analysis was performed considering as outcome the dichotomous classification of PSEQ-I score.

All tests will be considered significant with *p* values less than 0.05 and 95% confidence intervals were provided for variables subjected to statistical inference. All statistical analyses were performed using R version 3.4.1 for Windows (The R foundation for Statistical Computing).

## Results

### Participants

The medical records of 310 outpatients with chronic non-specific LBP afferent to three different physical therapy clinics were retrospectively analyzed. 165 out of 310 patients participated also to the sub-analysis on lumbar disability, since only data from the study by Chiarotto and colleagues [15] included the measure of lumbar disability by the RMDQ, whereas the other two databases used for this secondary analysis did not include that measure.

### Descriptive data

Table 1 shows the socio-demographic and clinical characteristics of the sample, whereas Table 2 illustrates mean and SD of pain self-efficacy, amount of pain and disability (RMDQ scores concerned 165 subjects). 199 subjects (64.2% of the sample) did not reach 40 points score, showing poor self-efficacy.

**Table 1:** Socio-demographic characteristics of the study population (*n* = 310)

Variable		*n* = 310
AGE (mean (sd))		49.83 (14.35)
SEX (%)	Male	120 (38.7)
	Female	190 (61.3)
EDUCATION (%)	Elementary school	2 (0.6)
	Middle school	41 (13.3)
	High school	132 (42.7)
	University	134 (43.4)
	Not Available	1
SMOKING (%)	No	228 (73.5)
	Yes	82 (26.5)
WEIGHT [Kg] (mean (sd))		69.68 (13.42)
HEIGHT [cm] (mean (sd))		169.14 (9.40)
BMI^a^ (mean (sd))		24.24 (3.58)
PAIN DURATION [mos] (median [IQR])		12 [6, 36]
PAIN LOCALIZATION (%)	Lumbar pain	243 (78.4)
	Referred pain	67 (21.6)
DRUGS (%)	No	142 (45.8)
	Yes (NSAIDs, pain killers, etc)	168 (54.2)

^a^*BMI* Body mass index

**Table 2:** Rating scales of the study population (*n* = 310)

Variable	Levels	*n* = 310
Pain self-efficacy *(n* = 310)		
PSEQ-I (mean (sd))		34.63 (13.39)
PSEQ-I (%)	>40	111 (35.8)
	≤40	199 (64.2)
Pain (*n* = 310)		
NRS (mean (sd))		4.66 (1.86)
NRS 0 (%)	No Pain	0 (0.0)
NRS [1–3] (%)	Mild Pain	88 (28.7)
NRS [4–6] (%)	Moderate Pain	162 (52.8)
NRS [7–10] (%)	Severe Pain	57 (18.5)
	Not Avaliable	3
Disability (*n* = 165)		
RMDQ (mean (sd))		8.92 (4.75)
RMDQ [0–9]	Low Disability	96 (58.2)
RMDQ [10–13]	Intermediate Disability	40 (24.2)
RMDQ ≥14	High Disability	29 (17.6)

*NRS* Numerical rating scale, *PSEQ-I* Pain self-efficacy questionnaire – Italian version, *RMDQ* Roland & morris disability questionnaire

### Outcome data and main results

The relationships between PSEQ-I and the following demographic and clinical characteristics were investigated: age, gender, education, smoking, weight, height, Body Mass Index, localization and duration of LBP, and drugs use. Concerning the influence of these characteristics on poor self-efficacy, linear regression showed that having low PSEQ-I rates appeared significantly related to gender and drugs use. More specifically, being female determines PSEQ-I rate < 3.42 compared to being male (*p* = 0.028), and the drug use is related to a PSEQ-I rate < 5.15 (*p* = 0.001) compared to the not use. Multiple linear regression confirmed these results for gender (*p* = 0.05) and drugs use (*p* = 0.001). After having stratified the sample in PSEQ-I ≤ 40 and PSEQ-I > 40 rating, logistic regression confirmed these statistically significant results for gender (OR = 1.8, with *p* = 0.015) and drugs use (OR = 1.68, with *p* = 0.029).

Moreover, concerning the relationships between demographic and clinical characteristics and disability measured with RMDQ on 165 subjects, age (*p* = 0.02), being female (*p* = 0.018), low educational level (*p* < 0.001), height (*p* = 0.045), pain duration (*p* = 0.041), and drugs use (*p* < 0.001) are significantly related to higher disability. Multiple linear regression confirmed these results for age (*p* = 0.036), gender (*p* = 0.032), and educational level (*p* = 0.001).

Finally, concerning the relationships between demographic and clinical characteristics and the amount of pain measured with NRS, the following variables showed significant relationships with pain: educational level (*p* < 0.001), smoking (*p* = 0.021), height (*p* = 0.036), and drugs use (*p* < 0.001). Multiple linear regression confirmed these results for educational level (*p* = 0.001) and smoking (*p* = 0.013) (See Table 3 and Additional file 1: Tables S3.1, S3.2, S3.3, and S3.4).

**Table 3:** Univariate linear or logistic regression analysis

Linear regression models associated to PSEQ	Regression Coefficient	std.error	*p.*value	95% CIs		*R*^2^, %
Sex = female	−3.428	1.552	0.028	−6.481	−0.375	1.6
Drugs	−5.156	1.494	0.001	−8.097	−2.216	3.7
Logistic regression models associated to PSEQ > 40	Odds Ratio	std.error	*p.*value	95% CIs		AIC
Sex = female	1.8	0.242	0.015	1.121	2.899	404.4
Drugs	1.685	0.239	0.029	1.056	2.7	402.6
Linear regression models associated to NRS	Regression Coefficient	std.error	*p.*value	95% CIs		*R*^2^, %
Educational level [1–4]	−0.471	0.147	0.001	−0.76	−0.182	3.3
Smokers	0.56	0.24	0.021	0.087	1.033	1.7
Height [cm]	−0.026	0.012	0.036	−0.05	−0.002	1.7
Drugs	0.81	0.209	< 0.001	0.399	1.221	4.7
Linear regression models associated to RMDQ	Regression Coefficient	std.error	*p.*value	95% CIs		*R*^2^, %
AGE [yrs]	0.066	0.028	0.02	0.01	0.121	3.3
Sex = female	1.802	0.753	0.018	0.315	3.288	3.4
Educational level [1–4]	−1.684	0.458	< 0.001	−2.589	−0.779	7.6
Height [cm]	−0.08	0.04	0.045	−0.158	−0.002	2.4
Pain duration [mos]	0.011	0.005	0.041	0	0.021	2.6
Drugs	2.855	0.716	< 0.001	1.44	4.27	8.9

## Discussion

The aim of this study is two-fold: 1) to investigate the prevalence of pain self-efficacy, and 2) to inquire the relationships between pain self-efficacy, disability and pain on the one hand, and socio-demographic and clinical characteristics on the other, in chronic LBP.

Concerning the prevalence of pain self-efficacy, majority of our sample did not reach 40 points score, showing poor self-efficacy. A high prevalence of poor self-efficacy in chronic pain patients was also reported in a study on 1045 old veterans, among which 75% showed low to moderate self-efficacy levels [41]. Same percentage (75%) of poor self-efficacy was also found by Salvetti and colleagues [42] in a sample composed of 215 chronic LBP patients. Therefore, significant part of the chronic LBP patients do not use that significant protective factor, able to improve psychological resilience, by attenuating the consequences of pain intensity in terms of catastrophizing and depressive symptoms [43, 44].

The relationships with socio-demographic and clinical characteristics have never been investigated in Italian population, whereas the cross-sectional associations of pain self-efficacy with pain intensity and disability have been already inquired in a previous publication [15]. In that study, pain self-efficacy displayed moderate correlations with pain intensity (*r* = − 0.41) and disability (*r* = − 0.55). Association models adjusted for pain intensity also showed that pain self-efficacy was significantly and strongly associated with disability. Other studies showed that low pain self-efficacy was independently associated with greater functional disability [46], particularly in LBP patients [17, 27, 45]. In the present study, poor self-efficacy appeared significantly related to gender and drugs use, whereas we did not find any relevant correlations with age, according to the results of Rahman and colleagues [33] on chronic musculoskeletal pain patients, and those of Ahmed et al. [45] in LBP patients.

Contrary to Rahman and colleagues study [33], which did not show any significant association between pain self-efficacy and sex, we found that having poor pain self-efficacy and high disability appeared significantly related to female gender, according to the results of Jackson and colleagues [47] and Bartley and colleagues [48]. It is difficult to argue why women showed less pain self-efficacy score than men, because most of the literature concerning psychosocial issues (kinesiophobia, catastrophizing, fear-avoidance, etc.) in chronic LBP agree with more involvement in males [49–52].

The reasons for these different results can be related to sample characteristics: e.g. several studies were conducted on patients with severe pain, being under-represented subjects with mild or moderate pain. Moreover, cultural differences between countries can be implied, since most of other studies on psychosocial factors in chronic pain have been conducted on northern patients (from Netherlands, Sweden, Canada, etc.). This cultural difference may also include less self-confidence and less awareness on how many things Italian women do during the day. The female activities are often less valued than men ones outside of the home [53], and housework is not really considered as proper ‘work’ [54]. Therefore, less self-efficacy related to work performance may not be related to what the amount of work people do or do not do, but rather to their perceptions and to the values attributed to the various activities they perform [55].

Another explanation may be related to the PSEQ items, involving some activities differently performed by genders. For example, the item #2 [‘*I can do most of the household chores (e.g. tidying-up, washing dishes, etc.), despite the pain’]* and the item #5 [‘*I can do some form of work, despite the pain (“work” includes housework, paid and unpaid work’*] could be filled in correctly by women, but could be over- or underestimated by men, that usually do not perform housework. In addition, the answers to the item #3 [‘*I can socialise with my friends or family members as often as I used to do, despite the pain’]* and the item #6 (‘*I can still do many of the things I enjoy doing, such as hobbies or leisure activities, despite pain’*) could be influenced by other social differences between genders in Italian culture. As reported by Bartley and colleagues [48], ‘*feminine role cues may alter pain report more so than masculine role cues’.*

Concerning the relationship between gender and disability, another previous study reported more restriction in housework by females compared to men [56]. Sex differences in pain perception and self-efficacy include sex hormones, endogenous opioid function, genetic factors, coping strategies, catastrophizing, gender roles, and ways to describe pain [48]. In women, also tiredness, stress, interference and life dissatisfaction may influence psychological reaction to pain [49] and Italian women showed some difficulty in distinguishing between sensory and emotional information [57]. As reported by Roelofs and colleagues [50], *“the finding that male patients have somewhat (fear-avoidance beliefs) higher scores than female patients contradict research showing that female patients generally display higher levels of somatic and anxiety symptoms compared with male patients*”.

The second characteristics significantly related to self-efficacy in the present study is the drugs use. A significant relationship between passive coping (e.g. the use of a drug to manage pain) and less self-efficacy [58], higher pain and disability is quite logical. Who practices maladaptive coping styles avoids a stressful condition, disengages from stressful relationships, and uses or abuses of drugs and/or alcohol [59]. Tetsunaga and colleagues [60] suggested that the dominant factors influencing drug dependence were pain catastrophizing and disability. On the contrary, active coping strategies and higher self-efficacy involved less drugs use [61].

In the present study, disability (measured on 165 out of 310 patients) was also related to the higher age. Other studies found that disability in chronic LBP patients increases with advancing age [62], but indicators of quality of life are equal or even higher in older compared to younger patients [63]. Indeed, older patients experience more limitations within self-care/mobility and walking, but less problems with transportation compared to younger patients. Moreover, older or middle-aged LBP patients perceive more facilitation through architecture and products for communication, health services, social services and products for mobility than younger patients [64].

Educational level appeared significantly related to both disability and amount of pain.

Lower educational level has been identified as a factor associated with persistence of chronic widespread pain in a community study [65] *and* subjective disability was predicted by education [66]. Nevertheless, in agreement with a previous study [47], we did not find any correlation between educational level and pain self-efficacy. This implies that higher cultural level could not significantly increase pain self-efficacy; whereas, patients that are more educated seemed more likely improve their pain biology knowledge, after a pain education session [67].

The last characteristic significantly related to the amount of pain is smoking. Literature showed that current and former smokers have a higher prevalence and incidence of LBP than never smokers, despite this association is modest [68]. Smoking is also related to higher pain intensity [69] and is sometimes associated to a sedentary lifestyle, which is another risk factor for chronic LBP [68, 69].

Our study is the first one investigating the relationship between self-efficacy, pain, disability, and sociodemographic and clinical characteristics in an Italian population complained on chronic LBP. Since a reduced self-efficacy and increased fear avoidance are related to higher disability, a clinician may be interested in knowing the main characteristics potentially influencing pain self-efficacy.

The main limitation of this study is the generalization of the results to all Italian patients with chronic LBP, since our sample was composed of subjects with medium-high level of instruction, residents in northern Italy. Moreover, the dimension of the sample may have influenced the results. Further studies are suggested on the relationship between gender and self-efficacy, since the results of this retrospective study on Italian population are different from those performed in different counties.

## Conclusions

This study showed that the majority of our sample, composed of Italian people complained of chronic LBP, exhibits poor self-efficacy. Female gender and drugs use are significantly related to less self-efficacy and higher disability, low educational level negatively influences the amount of perceived pain and disability, and older age and smoking are related to disability and pain intensity, respectively.

From a clinical point of view, the knowledge of main sociodemographic and clinical characteristics potentially influencing chronic LBP may be useful to address more efforts towards the most negatively impacted subjects, among the entire population complained of chronic LBP.

## Supplementary information

**Additional file 1: Table S3.1.** Univariate linear regression association analysis with PSEQ. **Table S3.2.** Univariate logistic regression association analysis with PSEQ. **Table S3.3.** Univariate linear regression association analysis with NRS. **Table S3.4.** Univariate linear regression association analysis with RMDQ.

## Data Availability

The datasets used and/or analyzed during the current study are available from the corresponding author on reasonable request.

## Abbreviations

IQR: Interquartile range
LBP: Low Back pain
NRS: Numerical Rating Scale
OR: Odds Ratio
PSEQ-I: Pain Self-Efficacy Questionnaire – Italian version
RMDQ: Roland & Morris Disability Questionnaire
SD: Standard Deviation
STROBE: Strengthening the Reporting of Observational Studies in Epidemiology
