# Supplementary material for: Is there a relationship between self-efficacy, disability, pain and sociodemographic characteristics in chronic low back pain? A multicenter retrospective analysis
Source: Arch Physiother. 2019 Oct 12;9:9. doi: 10.1186/s40945-019-0061-8 (PMC6790029; doi:10.1186/s40945-019-0061-8)
Supplement: Supplementary file 1 — Additional file 1: Table S3.1. Univariate linear regression association analysis with PSEQ. Table S3.2. Univariate logistic regression association analysis with PSEQ. Table S3.3. Univariate linear regression association analysis with NRS. Table S3.4. Univariate linear regression association analysis with RMDQ. [file 40945_2019_61_MOESM1_ESM.docx]

**Table s3.1: Univariate linear regression association analysis with PSEQ**

| **PSEQ** | **Regression**  **Coefficient** | **std.error** | **p.value** | **95% CIs** | | **R^2^, %** |
| --- | --- | --- | --- | --- | --- | --- |
| ***AGE [yrs]*** | **-0.028** | **0.053** | **0.595** | **-0.133** | **0.076** | **0.1** |
| ***Sex = female*** | **-3.428** | **1.552** | **0.028** | **-6.481** | **-0.375** | **1.6** |
| ***Educational level [1-4]*** | **1.967** | **1.064** | **0.066** | **-0.128** | **4.061** | **1.1** |
| ***Smokers*** | **-1.937** | **1.723** | **0.262** | **-5.329** | **1.454** | **0.4** |
| ***Weight [kg]*** | **0.057** | **0.06** | **0.342** | **-0.061** | **0.176** | **0.3** |
| ***Height [cm]*** | **0.042** | **0.086** | **0.624** | **-0.127** | **0.212** | **0.1** |
| ***BMI [Kg/m^2^]*** | **0.199** | **0.226** | **0.38** | **-0.246** | **0.645** | **0.3** |
| ***Referred pain*** | **-1.797** | **1.848** | **0.332** | **-5.433** | **1.839** | **0.3** |
| ***Pain duration [mos]*** | **0.018** | **0.013** | **0.191** | **-0.009** | **0.044** | **0.6** |
| ***Drugs*** | **-5.156** | **1.494** | **0.001** | **-8.097** | **-2.216** | **3.7** |

**Table s3.2: Univariate logistic regression association analysis with PSEQ**

| **PSEQ >40** | **Odds Ratio** | **std.error** | **p.value** | **95% CIs** | | **AIC** |
| --- | --- | --- | --- | --- | --- | --- |
| ***AGE [yrs]*** | **1.003** | **0.008** | **0.747** | **0.987** | **1.019** | **404.4** |
| ***Sex = female*** | **1.8** | **0.242** | **0.015** | **1.121** | **2.899** | **404.4** |
| ***Educational level [1-4]*** | **0.775** | **0.17** | **0.135** | **0.552** | **1.078** | **403.5** |
| ***Smokers*** | **0.889** | **0.267** | **0.66** | **0.529** | **1.509** | **404.4** |
| ***Weight [kg]*** | **0.991** | **0.009** | **0.317** | **0.972** | **1.009** | **349.0** |
| ***Height [cm]*** | **0.992** | **0.014** | **0.558** | **0.966** | **1.019** | **349.0** |
| ***BMI [Kg/m^2^]*** | **0.971** | **0.035** | **0.415** | **0.906** | **1.042** | **349.0** |
| ***Referred pain*** | **1.086** | **0.29** | **0.776** | **0.62** | **1.94** | **404.4** |
| ***Pain duration [mos]*** | **0.999** | **0.002** | **0.65** | **0.995** | **1.003** | **401.5** |
| ***Drugs*** | **1.685** | **0.239** | **0.029** | **1.056** | **2.7** | **402.6** |

**Table s3.3: Univariate linear regression association analysis with NRS**

| **NRS** | **Regression**  **Coefficient** | **std.error** | **p.value** | **95% CIs** | | **R^2^, %** |
| --- | --- | --- | --- | --- | --- | --- |
| ***AGE [yrs]*** | **0.007** | **0.007** | **0.317** | **-0.007** | **0.022** | **0.3** |
| ***Sex = female*** | **0.325** | **0.218** | **0.136** | **-0.103** | **0.753** | **0.7** |
| ***Educational level [1-4]*** | **-0.471** | **0.147** | **0.001** | **-0.76** | **-0.182** | **3.3** |
| ***Smokers*** | **0.56** | **0.24** | **0.021** | **0.087** | **1.033** | **1.7** |
| ***Weight [kg]*** | **-0.006** | **0.009** | **0.489** | **-0.023** | **0.011** | **0.2** |
| ***Height [cm]*** | **-0.026** | **0.012** | **0.036** | **-0.05** | **-0.002** | **1.7** |
| ***BMI [Kg/m^2^]*** | **0.023** | **0.033** | **0.48** | **-0.041** | **0.088** | **0.2** |
| ***Referred pain*** | **-0.418** | **0.258** | **0.106** | **-0.925** | **0.09** | **0.9** |
| ***Pain duration [mos]*** | **-0.001** | **0.002** | **0.764** | **-0.004** | **0.003** | **0.2** |
| ***Drugs*** | **0.81** | **0.209** | **<0.001** | **0.399** | **1.221** | **4.7** |

**Table s3.4: Univariate linear regression association analysis with RMDQ**

| **RMDQ** | **Regression**  **Coefficient** | **std.error** | **p.value** | **95% CIs** | | **R^2^, %** |
| --- | --- | --- | --- | --- | --- | --- |
| ***AGE [yrs]*** | **0.066** | **0.028** | **0.02** | **0.01** | **0.121** | **3.3** |
| ***Sex = female*** | **1.802** | **0.753** | **0.018** | **0.315** | **3.288** | **3.4** |
| ***Educational level [1-4]*** | **-1.684** | **0.458** | **<0.001** | **-2.589** | **-0.779** | **7.6** |
| ***Smokers*** | **0.005** | **0.889** | **0.996** | **-1.751** | **1.761** | **0.2** |
| ***Weight [kg]*** | **-0.014** | **0.029** | **0.631** | **-0.072** | **0.044** | **0.1** |
| ***Height [cm]*** | **-0.08** | **0.04** | **0.045** | **-0.158** | **-0.002** | **2.4** |
| ***BMI [Kg/m^2^]*** | **0.098** | **0.1** | **0.329** | **-0.1** | **0.295** | **0.6** |
| ***Referred pain*** | **0.19** | **1.003** | **0.85** | **-1.79** | **2.17** | **0.2** |
| ***Pain duration [mos]*** | **0.011** | **0.005** | **0.041** | **0** | **0.021** | **2.6** |
| ***Drugs*** | **2.855** | **0.716** | **<0.001** | **1.44** | **4.27** | **8.9** |
